# *Jiang-Zhi* granules decrease sensitivity to low-dose CCl_4_ induced liver injury in NAFLD rats through reducing endoplasmic reticulum stress

**DOI:** 10.1186/s12906-019-2641-2

**Published:** 2019-08-22

**Authors:** Lili Yang, Yan Zhou, Haiyan Song, Peiyong Zheng

**Affiliations:** 1 Institute of Digestive Diseases, Longhua Hospital Shanghai University of Traditional Chinese Medicine, Shanghai, 200032 China; 2 China-Canada Centre of Research for Digestive Diseases, Shanghai, 200032 China

**Keywords:** NAFLD, Liver injury, *Jiang-Zhi* granules, CCl_4_, ERS

## Abstract

**Background:**

Non-alcoholic fatty liver disease (NAFLD) may increase the sensitivity to liver injury caused by stimulants such as drugs and poisons. The traditional Chinese medicine (TCM) *Jiang-Zhi* Granule (JZG) has been proven effective for improving liver function, reducing hepatic fat accumulation and inflammation in NAFLD. The purpose of this study is to evaluate the effect of JZG on the susceptibility of NAFLD rats to liver injury and to identify the relevant mechanism.

**Methods:**

Forty wistar rats were randomly divided into five groups, normal group, normal+CCl_4_ group, high-fat diet (HFD) group, HFD + CCl_4_ group, and HFD + CCl_4_ + JZG group. NAFLD were established with HFD for 8 weeks. Then Low-dose CCl_4_ was given intraperitoneally to induce liver injury in NAFLD rats for 48 h. From the 5th week of HFD, intragastric administration of JZG was simultaneously given to the rats in the HFD + CCl_4_ + JZG group. At the end of the experiment, liver histological pathology, serum transaminase, lipid in liver and blood, as well as hepatic expression levels of endoplasmic reticulum stress (ERS) related molecules were evaluated.

**Results:**

NAFLD rat model was established by eight-week HFD feeding, exhibiting elevated levels of hepatic lipid, blood lipid, serum transaminase and significantly increased expression of ERS related molecules including glucose regulating protein 78 (GRP78), protein kinase RNA-like endoplasmic reticulum kinase (PERK), eukaryotic translation initiation factor 2α (EIF2α), and nuclear factor-kappa B (NFκB) in liver tissues. After injection of CCl_4_ in NAFLD rats, elevated serum transaminases, severe inflammation and focal necrosis were observed in liver tissue, but no obvious change was found in the rats of normal group. JZG reduced hepatic inflammation, hepatic necrosis, hepatic lipid, blood transaminases and blood lipids in HFD + CCl_4_ rats. ERS related molecules were significantly elevated by low-dose CCl_4_ in NAFLD rats, and were down-regulated by JZG.

**Conclusion:**

The sensitivity to CCl_4_-induced liver injury is increased in NAFLD rats, which could be improved by JZG. The pharmacological mechanism may involve the regulation of ERS signaling pathway by JZG.

## Background

Along with the global prevalence of obesity and diabetes, the incidence of non-alcoholic fatty liver disease (NAFLD) is increasing rapidly. According to one meta-analysis, the recent global prevalence of NAFLD is up to 25.24% (95% CI: 22.10–28.65) [1]. The prevalence of non-alcoholic steatohepatitis (NASH) in NAFLD patients who had liver biopsy with a specific “clinical indication” is estimated to be 59.10% (95% CI, 47.55–69.73). At the stage of NASH, the risk of liver cirrhosis, hepatocellular carcinoma, and liver failure increases dramatically and might eventually leads to death [2]. At present, NASH has become the second largest risk factor for adult liver transplantation [3]. Compared with normal individuals, patients with NAFLD have a higher mortality rate and risk of developing cardiovascular disease or metabolic syndrome related tumors [4, 5].

Previous researches have reported that liver sensitivity to acute drug or toxin induced injury was increased in NAFLD [6–8]. For example, after intraperitoneal injection of CCl_4_, the mortality rate of the NAFLD rats induced by methionine and choline-deficient diet reached70% within 12-72 h, whereas all the normal control rats survived, indicating markedly increased sensitivity of NAFLD rats to CCl_4_-induced liver injury [9].

The pathogenesis of NAFLD has not yet been comprehensively elucidated, although the “multi-hit” hypothesie is generally accepted [10]. Recent studies have confirmed that many liver diseases, including NAFLD, are associated with endoplasmic reticulum stress (ERS) [11–14]. Endoplasmic reticulum (ER) is the site of protein synthesis, folding, transit and modification. ER is extremely sensitive to various stimuli, including hypoxia, high glucose, chemical poisons and other factors, which may lead to accumulation of misfolded and unfolded proteins in the lumen of ER and causing ERS. The first reaction that occurs in ERS is the up-regulation of molecular chaperone glucose regulating protein 78 (GRP78) to improve protein folding or to correct misfolding.. However, the binding of GRP78 and unfolded proteins results in its dissociation with the stress sensor protein kinase RNA-like endoplasmic reticulum kinase (PERK) and inositol requiring enzyme 1α (IRE1α), etc., which cause the activation of these proteins and triggering ERS. After the dissociation of PERK and GRP78, PERK is activated by dimerization and autophosphorylation, which phosphorylates the downstream factor eukaryotic translation initiation factor 2α (EIF2α), thereby forming P-EIF2α. P-EIF2α regulates the activation of NFkB via reducing the synthesis of inhibitor of NFκB (IκB) [14–16]. When IRE1α was activated, IкB kinase (IKK) was recruited by tumor necrosis factor receptor associated factor 2 (TRAF2), which promotes NFκB mediated inflammation [17, 18].

*Jiang-Zhi* Granule (JZG), composed of *Gynostemma pentaphyllum (Thunb.) Makino*, *Polygoni cuspidati* rhizoma, *Artemisia capillaris Thunb*, *Salviae Miltiorrhizae* Radix, and *Folium Nelumbinis*, has been used to treat fatty liver and hyperlipidemia in clinic for a decade. In December 2008, JZG was approved by State Food and Drug Administration (SFDA) (Approval No: 2008 L11181) in a new drug clinical trial. One randomized and placebo-controlled multicenter clinical trials conducted by 6 national GCP bases showed an increased liver/spleen CT mean value in the JZG group by 0.26 ± 0.23 above baseline, compared with the placebo group showing a growth of 0.07 ± 0.22. The effective rate of JZG group was 71.19% while the placebo group was 42.57% [19]. In in vivo experiments, JZG improved hepatosteatosis and liver injury in NAFLD rat model induced by high-fat diet (HFD) [20]. And in vitro experiments also suggest that JZG could alleviate the lipid deposition and injury in hepatocytes induced by fatty acid [20, 21].

Therefore, the objective of this study is to evaluate the effect of JZG on the sensitivity to liver injury in NAFLD rats. In addition, since ERS is closely related to NAFLD and liver injury, the ERS signaling pathway was evaluated to explore the molecular mechanism of JZG.

## Methods

### Animals and interventions

Forty male wistar rats, body weight 160 ± 10 g, purchased from Beijing Vital River Laboratory Animal Technology Co., Ltd., were randomly divided into normal group, normal+CCl_4_ group, HFD group, HFD + CCl_4_ group and HFD + CCl_4_ + JZG group (*N* = 8/group) after one-week adaptive feeding. The normal group and the normal+CCl_4_ group were given ordinary diet and the others were given HFD for 8 weeks (HFD consists of protein, fat and carbohydrate, with 20, 40 and 40% kcal%, respectively). All animals were free to water and food consumption. Rats were weighed once a week. Room temperature was kept at 24 °C ± 2 °C, humidity at 55 ± 10% and the illumination time for 12 h (8:00–20:00).

Oral intragastric administration of JZG was given to HFD + CCl_4_ + JZG group from the 5th week. After 8 weeks, rats in all groups except the normal group and the HFD group were given intraperitoneal injection with CCl_4_ at the dose of 0.27 g/kg. After 48 h, blood and liver tissue samples were collected under pentobarbital sodium at the dose of 0.11 g/kg anesthesia following a 12-h fast and then sacrificed via overdose of anesthetic.

All animal procedures were approved by the Animal Experiment Ethics Committee of Shanghai University of TCM (No. SZY201504024).

### Drug preparation

The components of JZG included *Gynostemma pentaphyllum (Thunb.) Makino* (15 g), *Polygoni cuspidati* rhizome (15 g), *Artemisia capillaris Thunb* (9 g), *Salviae Miltiorrhizae* Radix (9 g), and *Folium Nelumbinis* (6 g), which were purchased from Longhua Hospital Shanghai University of Traditional Chinese Medicine. Its preparation method is based on the traditional Chinese medicine decocting method [22]. JZG was administered at the dose of 5.4 g/kg/d, according to the dose-equivalence equation between rats and humans [23].

### Staining of liver tissue

HE staining: liver tissue was fixed in 4% neutral formaldehyde solution overnight. The tissues were embedded with paraffin after dehydration and cut into 4 μm sections for HE staining. The stained section was photographed under an optical microscope.

Oil red O staining: the frozen liver tissue sections were fixed with 4% neutral formaldehyde solution for 30 min, then incubated for 30 min with oil red O staining solution and immersed for 15 s in 60% isopropyl alcohol. The section was rinsed with PBS twice, stained with hematoxylin for 1 min and then photographed under an optical microscope.

### Biochemical analysis

Alanine aminotransferase (ALT), aspartate aminotransferase (AST), albumin (ALB), γ-Glutamine transpetidase (γ-GT), triglyceride (TG), total cholesterol (TC), high density lipoprotein cholesterol (HDL-c), and low density lipoprotein cholesterol (LDL-c), were all tested with HITACHI 7170S automatic biochemical analyzer (Japan).

TG concentration in rat liver was assayed using commercial kits obtained from Nanjing Jiancheng Bioengineering Institute. A total of 200 mg liver tissues were homogenized in 3 mL of ethanol-acetone mixture (v: v = 1: 1) on ice for 20 s (repeat 2–3 times), and then stored overnight at 4 °C. After centrifugation at 3000 g/min at 4 °C for 20 min, the supernatant was analyzed for hepatic TG content following the manufacturer’s instruction.

### Real-time PCR for mRNA analysis

Liver tissue was homogenized in 1 ml TRIzol using MP-Fast homogenizer, and the total RNA was extracted. cDNA was obtained by reverse transcription with GoScript™ reagents and its amplification reaction was performed using Power SYBR Green PCR Master Mix (Applied Biosystems Foster City, CA, USA), with β-actin as the internal reference gene. Reaction conditions: denaturation at 95 °C for 10 min; 95 °C for 15 s, 60 °C for 1 min and 72 °C for 30s, which was repeated for 40 cycles. Relative gene expression was calculated with the 2^-ΔΔCT^ method [24]. Primer sequences were shown in Table 1.

**Table 1:** List of primers

Gene	Forward primer	Reverse primer
GRP78	GACTGGAATCCCTCCTGCTC	GGTCAGGCGGTTTTGGTC
PERK	TGGTAAAGTCATCCCCATCAGTC	CCTTGTAGGGAACTTTTCCGAG
EIF2α	CTGGGACCCCTAACCTACAAC	CATCTGACCAGGAAGGACACC
NF-kB	CTGCTTACGGTGGGATTGC	TGTTTCTTTCTCAGGGGGATTC
IRE1α	GAGGAATTACTGGCTTCTCATAGG	TTCTCGATGTTTGGGAAGATTG
TRAF2	TCCACCTATGATGGGGTCTTC	GCCGTCGCCATTCAAGTAG
β-actin	CCCATCTATGAGGGTTACGC	TTTAATGTCACGCACGATTTC

### Protein isolation and western blotting

Liver tissue was homogenized in RIPA lysis buffer containing protease inhibitor (Complete mini EASY pack, Roche, Basel, Swiss) to extract protein. The concentration was determined by BCA kit (CoWin Bioscience, Beijing, China) and protein was resolved with 10% polyacrylamide gel, then transferred to the PVDF membrane (Millipore, Billerica, MA, USA). After blocking, they were incubated overnight in antibody dilutions of GRP78 (1:1000) (CST, Boston, MA, USA, 3183), P-PERK (1:1000) (CST, Boston, MA, USA, 3179), P-EIF2α (1:1000) (CST, Boston, MA, USA, 3398), P-NFκB (1:500) (CST, Boston, MA, USA, 3033), P-IREα (Abcam, Cambridge, UK, ab148187), TRAF2 (1:1000) (Abcam, Cambridge, UK, ab126758) or β-actin (1:1000) (Huaan biological technology, Hangzhou, China), and secondary antibody were incubated for 2 h at room temperature. The images of blots were acquired by GBOX Chemi XT4 gel imaging system (Syngene, Cambridge, UK).

### Statistical analysis

Statistical analysis and graphing were conducted with the SPSS 18.0 and GraphPad Prism 6.0 software. Data were expressed as mean ± standard deviation (SD). One-way ANOVA was utilized in the comparison among different groups and Tukey’s post-hoc test was applied for comparison between two groups. *P < 0.05* was considered statistically significant difference.

## Results

### Effect of JZG on histopathology of liver tissue

**Fig. 1:**
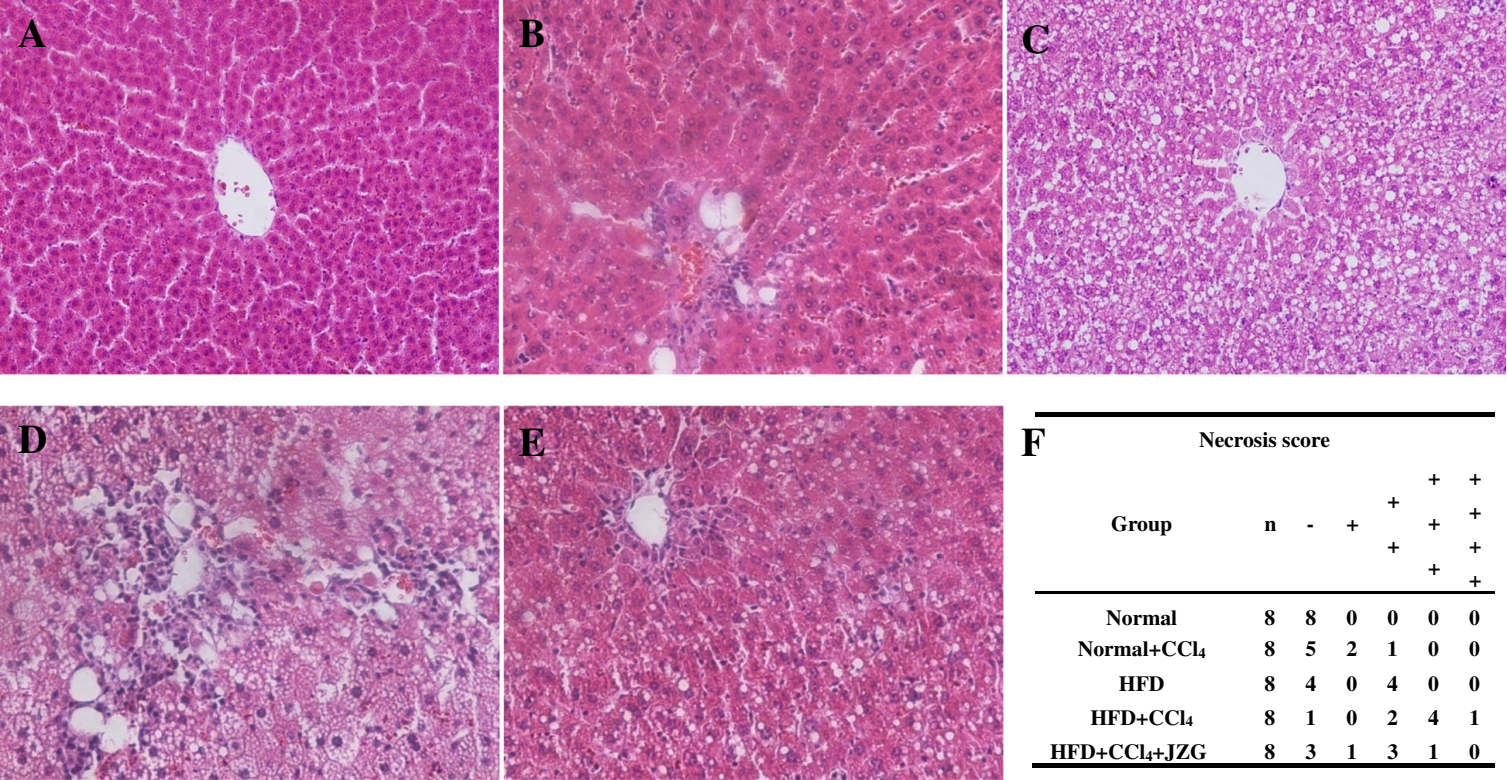
Effect of JZG on the histopathology of liver tissues of rat: **a**-**e** HE staining of liver tissue, the original magnification is 200×, **a** Normal group, **b** Normal+CCl_4_ group, **c** HFD group, **d** HFD + CCl_4_ group, **e** HFD + CCl_4_ + JZG group, **f** Necrosis score of liver histopathology

HE staining (Fig. 1) showed, the rat liver tissue of normal group was structurally complete without abnormal histological structure (Fig. 1a), while different-size lipid droplets were accumulated in hepatocytes of HFD rats (Fig. 1c). There was almost no change in normal+CCl_4_ group except mild diffuse of hepatocellular focal necrosis in three rats (Fig. 1b). In contrast, liver injury was more severe in HFD + CCl_4_ group (Fig. 1d) than HFD group, with infiltration of inflammatory cells and vacuolar degeneration around central vein as well as obvious localized focal necrosis besides severe steatosis. The inflammation, necrosis and hepatocyte vacuolar degeneration were alleviated obviously by JZG (Fig. 1e).

According to the counting of the necrotic foci under 200× microscope, five degrees of hepatocellular necrosis in pathological tissue were defined: “-”: no necrotic foci; “+”: less than 2 necrotic foci; “++”: 2 to 4 necrotic foci; “+++”: 4 to 10 necrotic foci; “++++”: more than 10 necrotic foci [18] (Fig. 1f).

### Effect of JZG on liver function

**Fig. 2:**
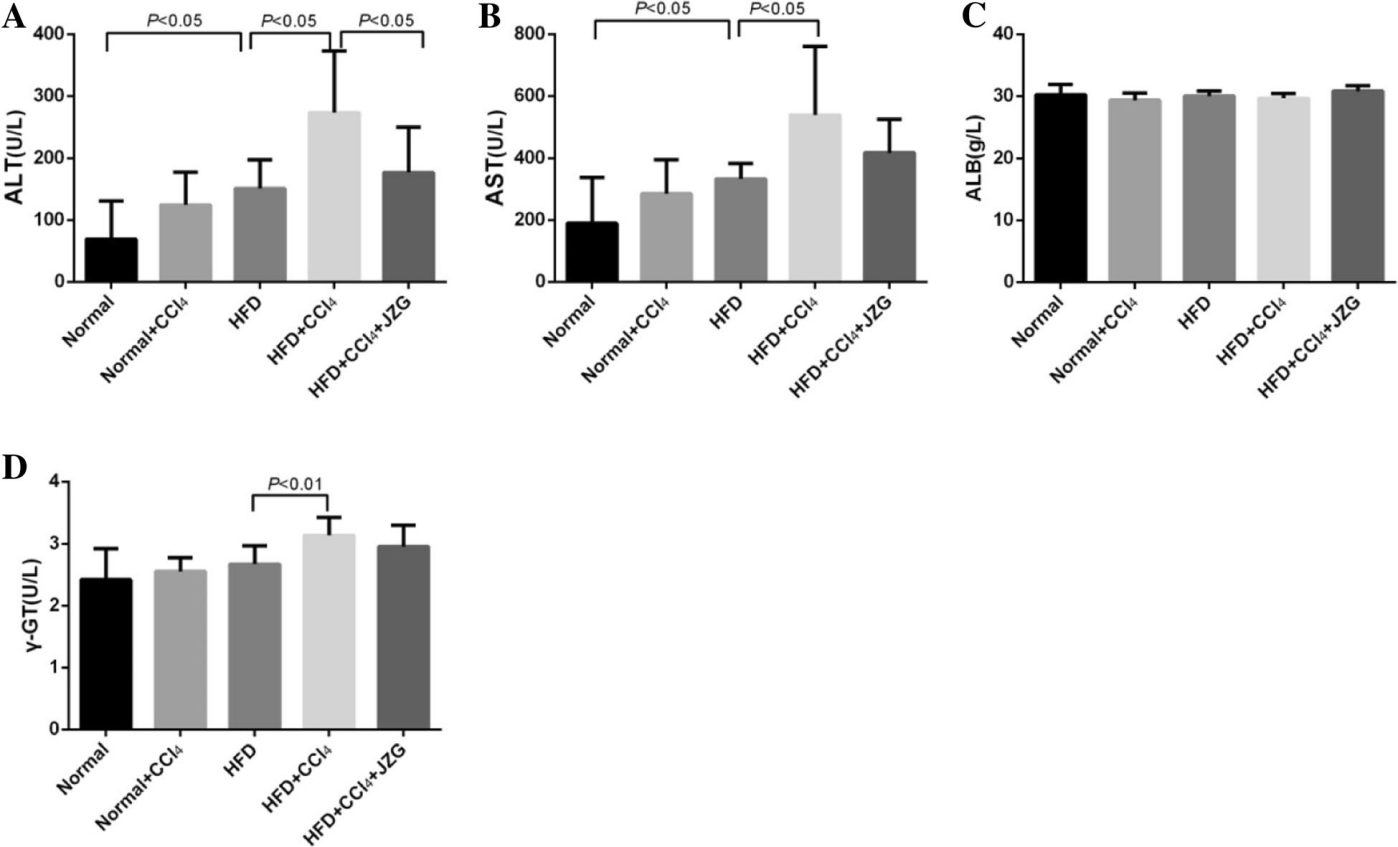
Effect of JZG on liver function: **a** The serum ALT level, **b** The serum AST level, **c** The serum ALB level, **d** The serum γ-GT level. The values represent the mean ± SD. (*N* = 8 per group

Serum ALT and AST levels in HFD group were significantly higher than those in normal group (*P* < 0.05), there was no statistically significant difference between normal group and normal+CCl_4_ group (*P* > 0.05). However, in comparison with rats in the HFD group, the levels of serum ALT, AST and γ-GT in the HFD + CCl_4_ group were increased (*P* < 0.05). JZG apparently reduced the level of serum ALT in rats induced by HFD + CCl_4_ (*P* < 0.05), and have a tendency to reduce serum AST and γ-GT levels. The content of serum ALB was comparable in all study groups (*P > 0.05*) (Fig. 2). The results suggested that HFD and HFD + CCl_4_ could cause damage to the liver function of rats, and low-dose CCl_4_ in combination with HFD cause more serious liver dysfunction. JZG could improve rat liver function with damage induced by HFD + CCl_4_.

### Effect of JZG on hepatosteatosis

**Fig. 3:**
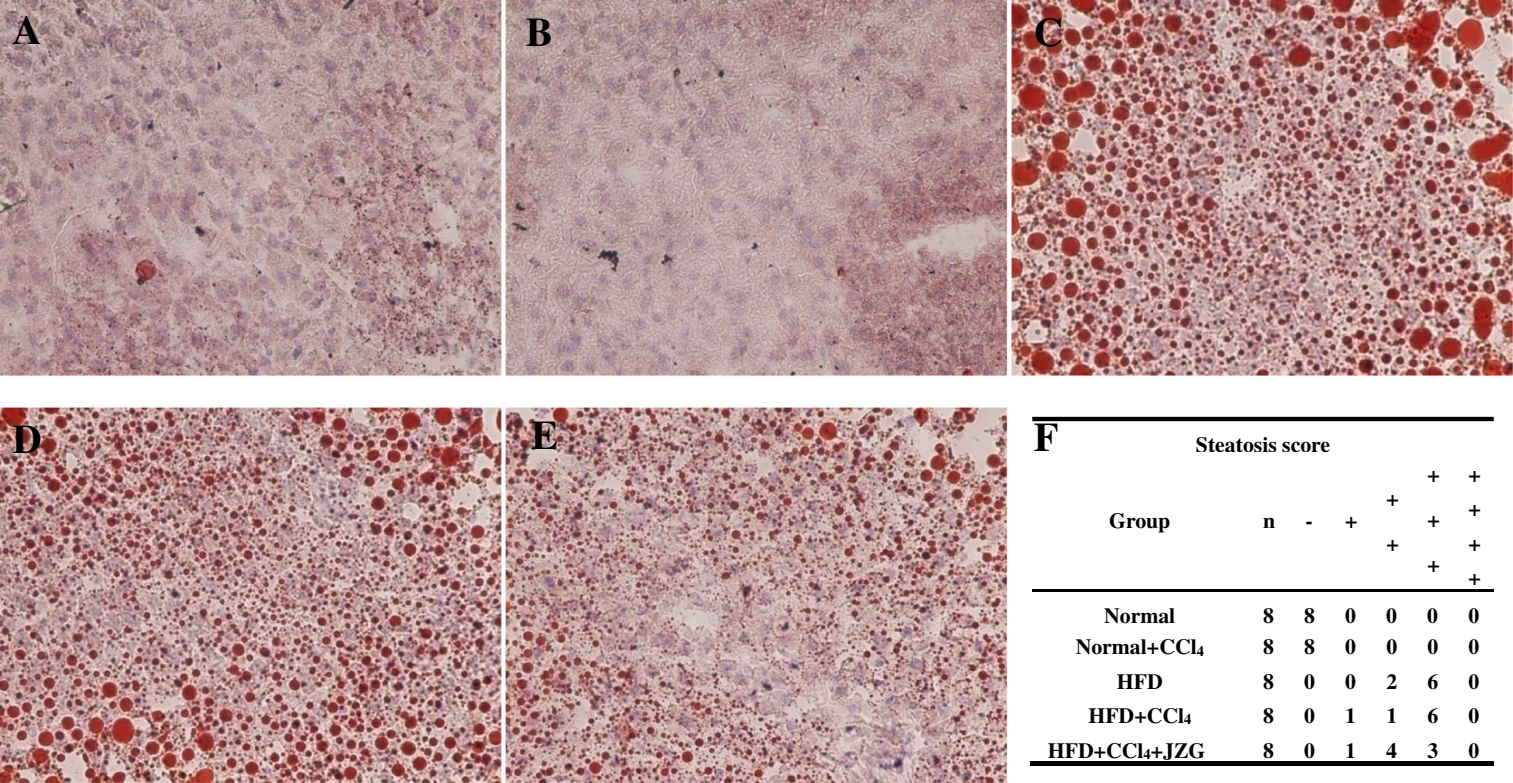
Effect of JZG on hepatosteatosis: **a**-**e** Oil Red O Staining in liver tissue, the original magnification is 200×, **a** Normal group, **b** Normal+CCl_4_ group, **c** HFD group, **d** HFD + CCl_4_ group, **e** HFD + CCl_4_ + JZG group, **f** Steatosis stage of histopathology

Oil red O staining demonstrated that there was no significant steatosis in liver tissue of rats in normal group or normal+CCl_4_ group (Fig. 3a-b). Extensive hepatocellular steatosis were observed in HFD group and HFD + CCl_4_ group (Fig. 3c-d), while JZG improved the steatosis in liver tissuse (Fig. 3e).

The steatosis was graded according to the percentage of hepatocellular steatosis of liver tissue: “-”: less than 5% hepatocellular steatosis; “+”: 5 to 30%; “++”: 31 to 50%; “+++”: 51 to 75%; “++++”: more than 75% [18] (Fig. 3f).

**Fig. 4:**
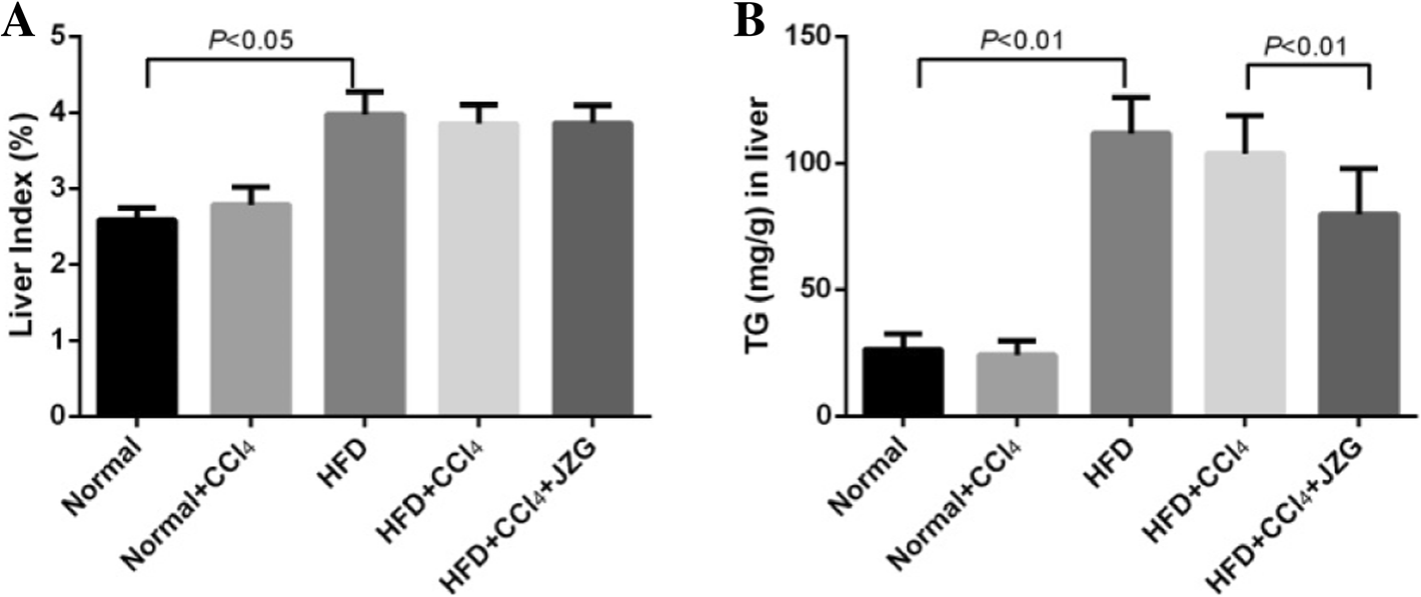
Effect of JZG on liver index and TG content in liver: **a** Liver index **b** Liver TG content. The values represent mean ± SD. (*N* = 8 per group)

Liver index of rats in the HFD group was markedly higher than that in normal group (*P < 0.05*), and JZG did not decrease the liver index (Fig. 4). The level of TG in rat liver induced by HFD increased dramatically when compared to the normal group (*P < 0.05*). There was no significant difference between HFD group and HFD + CCl_4_ group (*P > 0.05*), while JZG could apparently reduce the TG level (*P < 0.05*).

The results demonstrated that massive hepatosteatosis appeared in the rats induced by HFD, which was not aggravated by low-dose CCl_4_ stimulation. JZG could ameliorate the lipid accumulation in liver.

### Effect of JZG on serum lipid

**Fig. 5:**
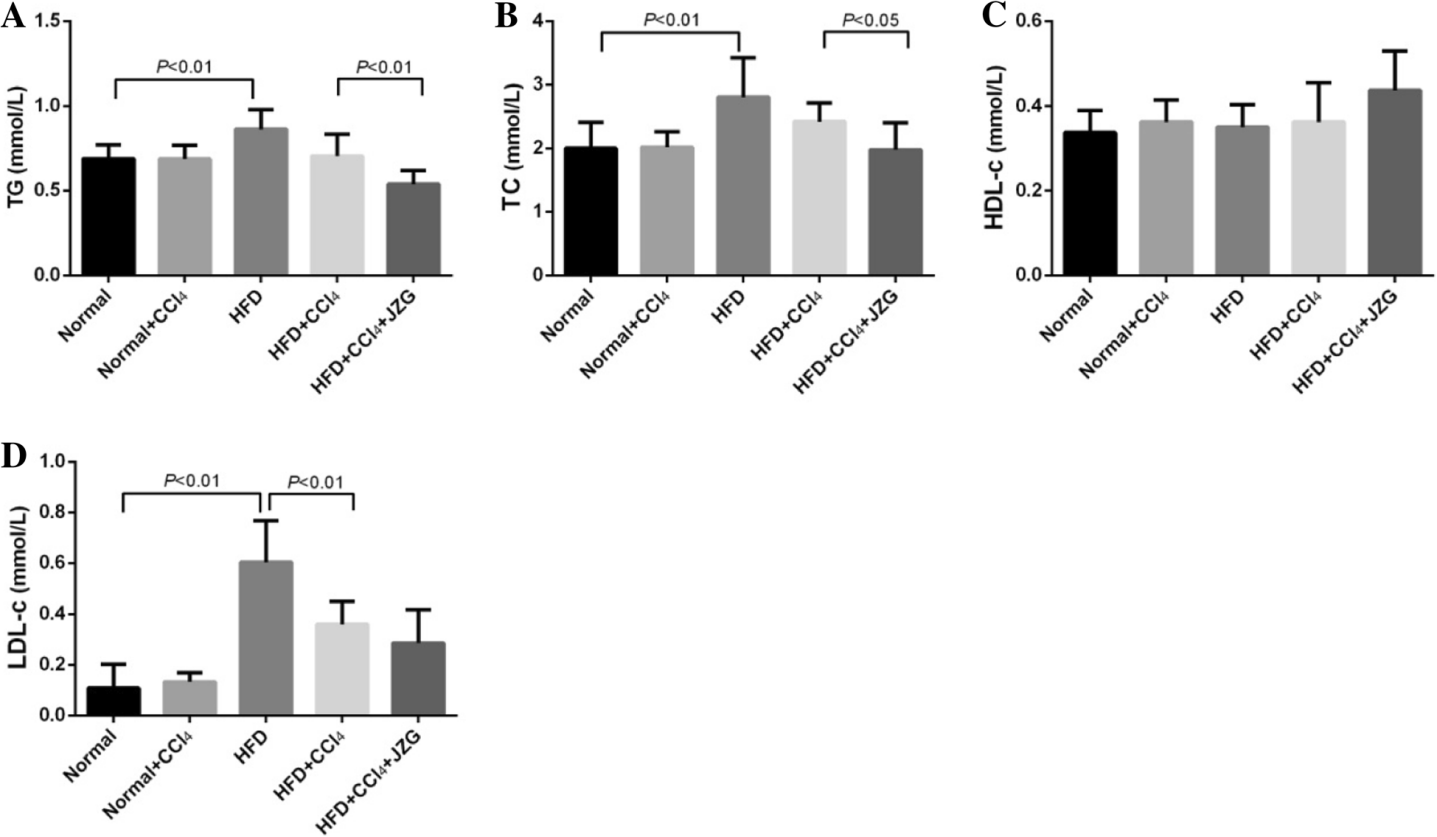
Effect of JZG on serum lipids: **a** The serum TG level, **b** The serum TC level, **c** The serum HDL-c level, **d** The serum LDL-c level. The values represent the mean ± SD. (*N* = 8 per group)

The results showed that the levels of TG, TC and LDL-c in rat serum increased markedly in the HFD group compared with the normal group (*P < 0.05*). There was no statistically significant difference between normal group and normal+CCl_4_ group (*P > 0.05*). In comparison with the HFD group, no obvious difference was demonstrated in rat serum TG, TC and LDL-c levels in the HFD + CCl_4_ group (*P > 0.05*). JZG decreased the rat serum TG and TC levels (*P < 0.05*) and had a tendency to reduce the level of LDL-c in the HFD + CCl_4_ group. The serum HDL-c level was comparable among different groups (*P > 0.05*) (Fig. 5). The results suggested that HFD could increase lipid contents in rat serum, while the low-dose CCl_4_ had no effect on the lipid level. JZG could improve the serum lipid levels in rat induced by HFD.

### Effect of JZG on ERS

**Fig. 6:**
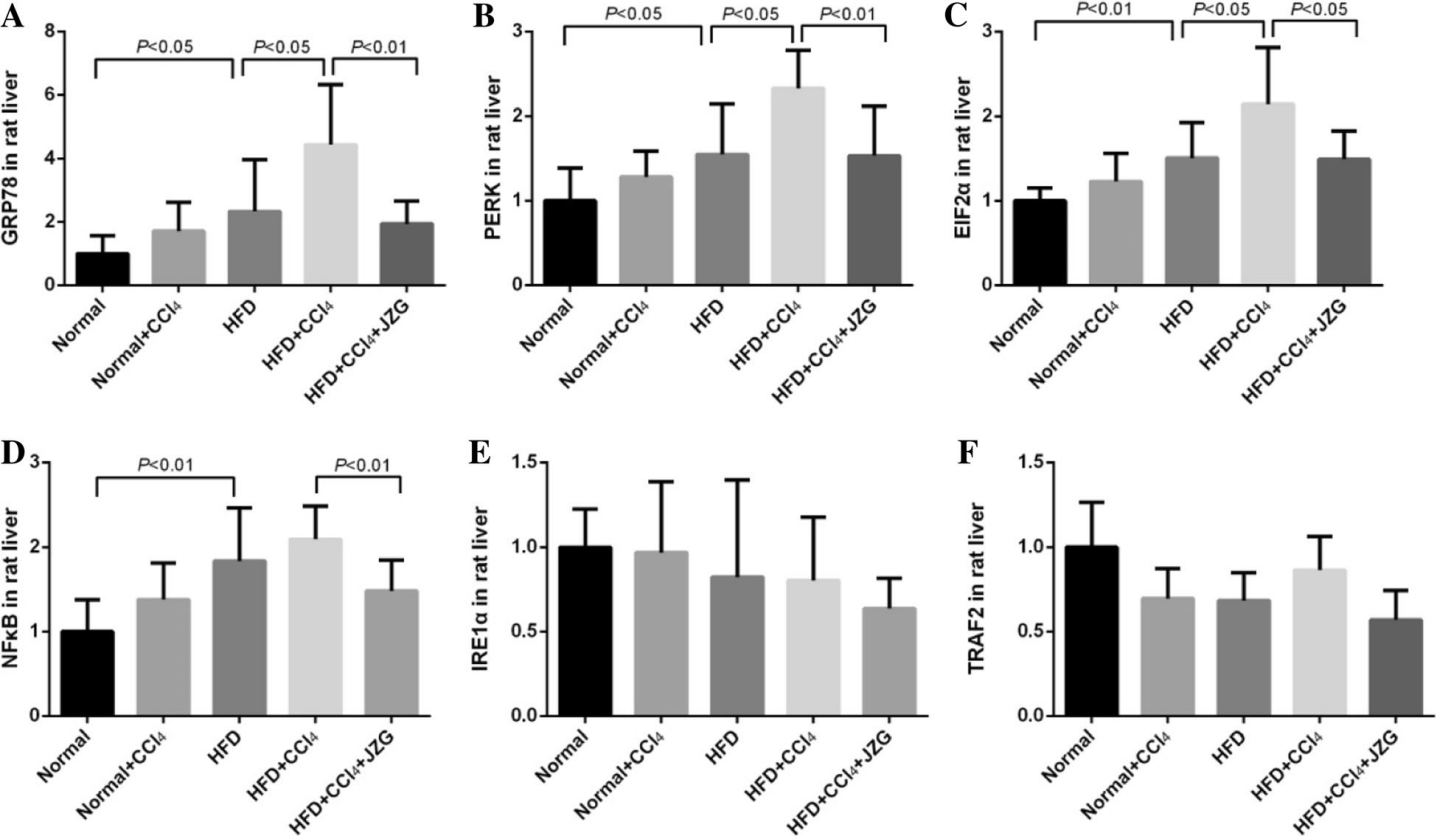
Effect of JZG on the mRNA levels of ERS related genes: **a** GRP78, **b** PERK, **c** EIF2α, **d** NFκB, **e** IRE1α, **f** TRAF2. The values represent mean ± SD. (*N* = 8 per group)

The mRNA levels of GRP78, PERK, EIF2α and NFκB in the liver of HFD group were up-regulated significantly compared to those of the normal group (*P < 0.05*). No difference was observed between the normal group and the normal+CCl_4_ group (*P > 0.05*), whereas marked increase in the expression of GRP78, PERK, EIF2α mRNA was observed in the HFD + CCl_4_ group than in the HFD group (*P < 0.05*). Treatment with JZG decreased the mRNA expression of GRP78, PERK, EIF2α and NFκB in rat liver tissues (*P < 0.05*) (Fig. 6). No significant difference was found in mRNA expression of IRE1α and TRAF2 among rats from different groups (*P > 0.05*).

**Fig. 7:**
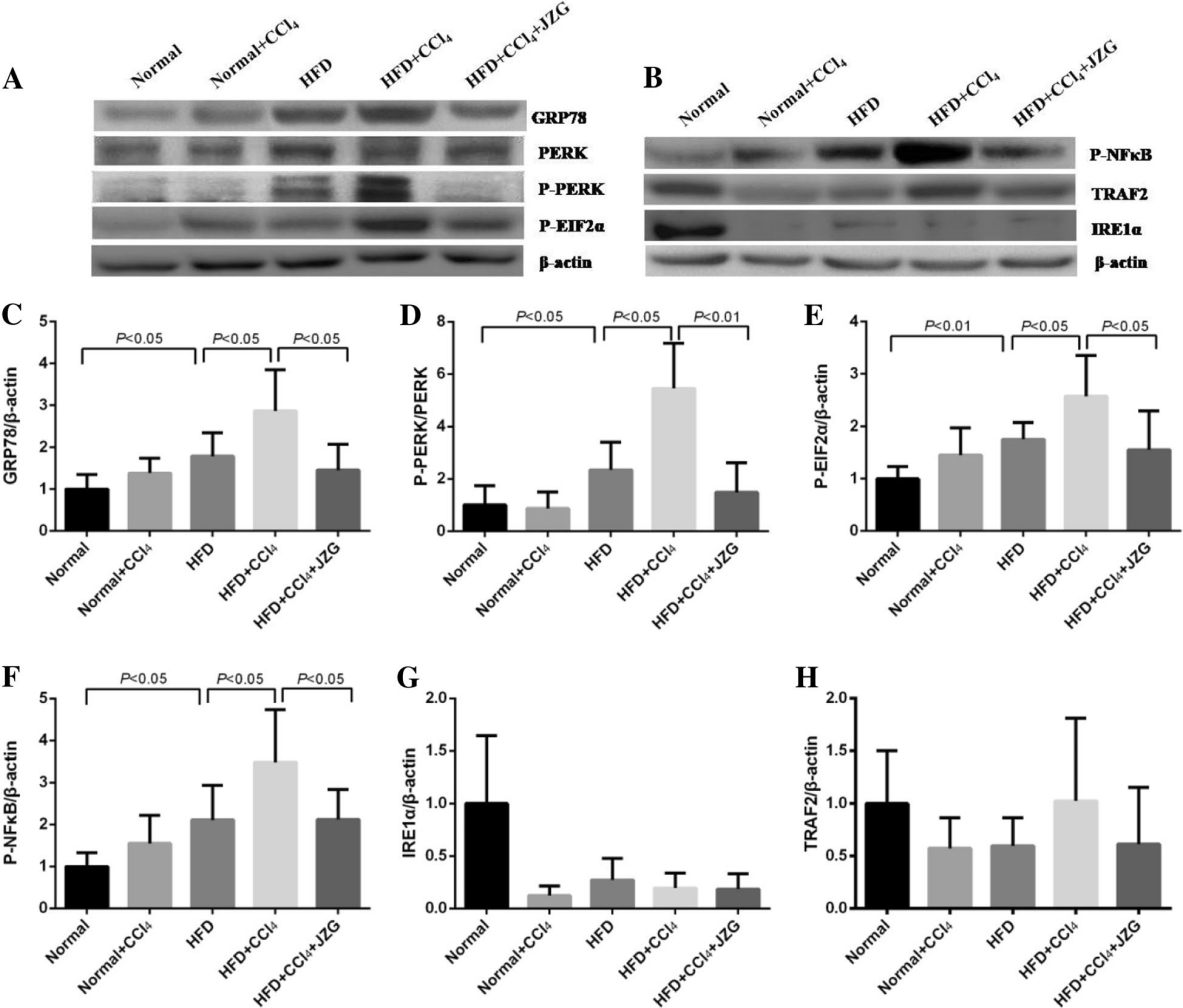
Effect of JZG on protein expression of ERS related genes: **a**-**b** Representative western blot results of GRP78, PERK, P-PERK, P-EIF2α, P-NFκB, IREα, and TRAF2 in rat liver tissues, **c**-**h** Densitometric analysis of GRP78, PERK, P-PERK, P-EIF2α, P-NFκB, IREα, and TRAF2, values were normalized to that of β-actin. The values represent the mean ± SD. (*N* = 6 per group)

Protein levels of GRP78, P-PERK, P-EIF2α and P-NFκB in the liver of the HFD group were increased significantly compared to those of the normal group (*P < 0.05*). No difference was observed between the normal group and the normal+CCl_4_ group (*P > 0.05*), whereas obvious increase were detected in the HFD + CCl_4_ group than in the HFD group (*P < 0.05*). JZG decreased the protein levels of GRP78, P-PERK, P-EIF2α and P-NFκB in rat liver tissues (*P < 0.05*). There was no significant difference in the protein expression of IRE1α and TRAF2 among different groups (*P > 0.05*) (Fig. 7).

The results suggested that HFD could induce ERS in liver, which was demonstrated by the significant increase of mRNA and protein expression of GRP78, PERK, EIF2α and NFκB. The low-dose CCl_4_ had less influence on normal rats, however, it could aggravate the ERS in HFD group, which may increase liver injury in NAFLD rats. JZG could reduce the levels of ERS related GRP78, PERK, EIF2α and NFκB mRNA and protein, mediating its mechanism in improving liver injury in NAFLD rats induced by low-dose CCl_4_.

## Discussion

NAFLD is a metabolic disease closely related to insulin resistance. Its disease spectrum includes non-alcoholic simple fatty liver (NAFL), NASH, and related hepatic cirrhosis and hepatocellular carcinoma (HCC) [25]. The incidence of NAFLD remains increasing [26], and there is a trend towards young people. Since NAFLD is asymptomatic or only with mildly elevated transaminase, most patients pay no attention to NAFLD and cannot receive effective treatment in time. In recent years, some studies have shown that NAFLD increases the risk of developing liver cirrhosis and cancer [27, 28]. Fan et al reported that the prevalence of obesity, hyperlipidemia, hypertension and diabetes in patients with NAFLD combined with Chronic Hepatitis B (CHB) was substantially higher than those of patients with simple CHB. During the 6.4 ± 3.5 years of follow-up, the mortality risk of HCC and liver disease in patients with NAFLD combined with CHB dramatically increased [29]. Therefore, patient with NAFLD is more sensitive to liver injury when combined with other harmful factors such as virus, alcohol, toxicant, etc. And it is important to study the mechanism of injury sensitivity and to explore the corresponding prevention measures.

Recent studies demonstrate that ERS plays an important role in the occurrence and development of NAFLD. ER is an important site for protein synthesis, post-translational modification and folding, with the function of lipid and carbohydrate synthesis, drug metabolism, storage and release of Ca^2+^ to maintain intracellular calcium homeostasis. ERS represents a state in which the internal and external environment of the cells changes, generating aggregation of unfolded proteins and the damage of calcium homeostasis in the ER lumen, thereby resulting in the state of ER dysfunction. High cholesterol and TG levels in NAFLD could induce ERS [30, 31], which could further aggravate the accumulation of cholesterol, ultimately leading to the vicious cycle in the pathogenesis of the NAFLD.

The NAFLD rat model with HFD was successfully established in this study. HE staining and oil red O staining demonstrated the existence of hepatosteatosis in rats of the HFD group compared with the normal group, and liver transaminases, blood lipids and liver lipids were significantly elevated. Low-dose CCl_4_ intraperitoneal injection had little impact on normal rats, while caused obvious liver injury in the HFD-induced NAFLD rats, as demonstrated by the apparent hepatocellular focal necrosis, numerous inflammatory cells infiltration in liver tissues, and significantly elevated aminotransferases compared to the HFD group. These indicated that the sensitivity to liver injury was increased in NAFLD rats. We further found that the mechanism of sensitivity to liver injury in NAFLD may be associated with the GRP78/PERK/EIF2α/NFκB signaling pathway in the ERS. The HFD + CCl_4_ group had an evident rise of the expression and activation of GRP78, PERK, EIF2α and NFκB in rats’ livers compared to the HFD group, suggesting that low-dose CCl_4_ could aggravate ERS in NAFLD rats.

**Fig. 8:**
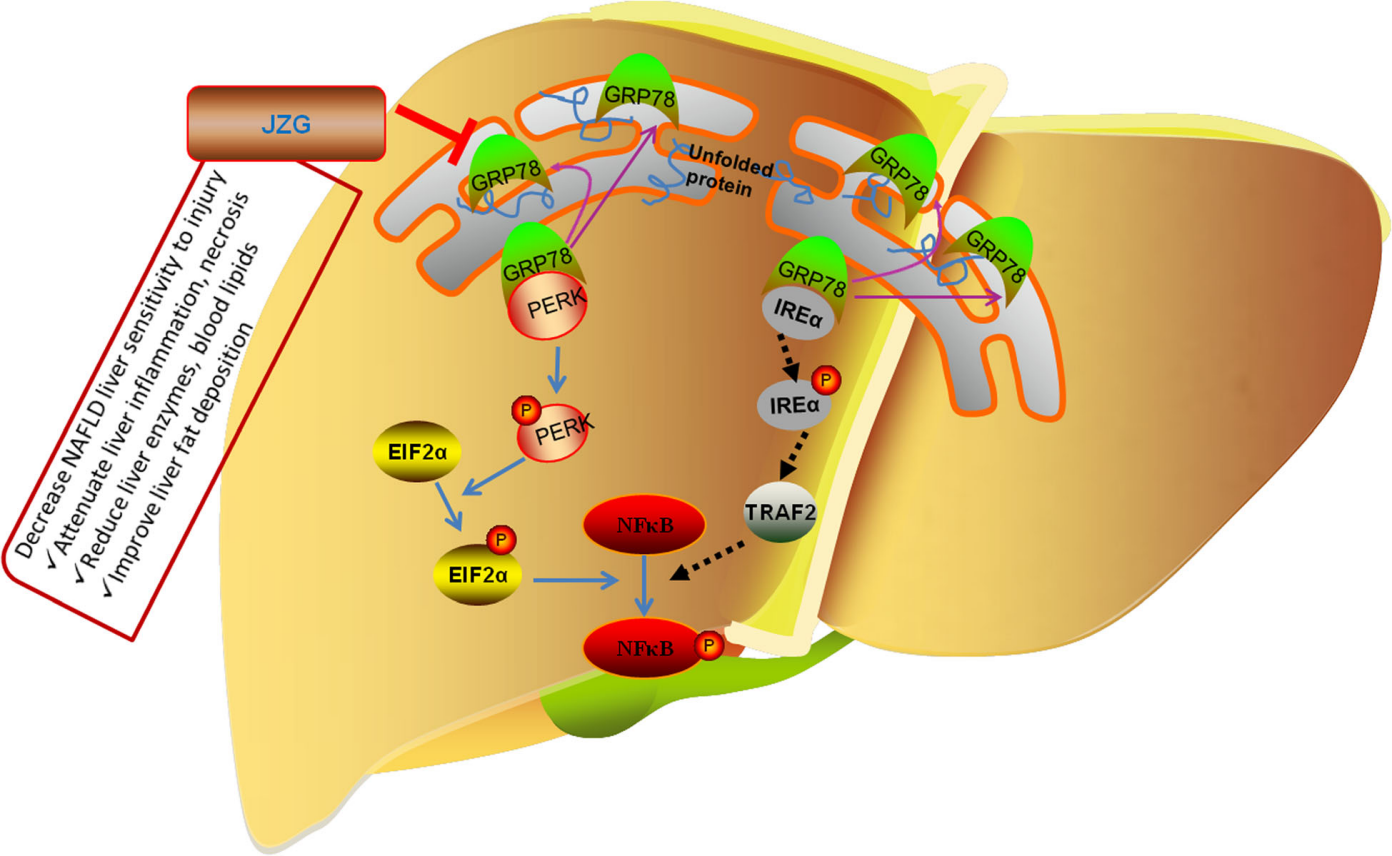
The schematic for JZG decreasing the sensitivity of NAFLD to liver injury. The schematic of proposed function mechanism for JZG was drew according to the results of this study. Various stimuli induce the accumulation of unfolded proteins in the ER and up-regulate the expression of GRP78 to improve the situation. However, the binding of GRP78 with unfolded proteins results in its dissociation with PERK and IRE1, causing the activation of these proteins and triggering ERS. PERK is activated by autophosphorylation and then phosphorylate EIF2α which promote the activation of NFκB. The activation of IRE1α could also mediate the further activation of NFкB through TRAF2. JZG could decrease the sensitivity of NAFLD to liver injury in rat through regulating ERS signaling pathway GRP78/PERK/EIF2α/NFκB, however, JZG did not affect the IRE1/TRAF/ NFκB pathway

To prevent severe liver injury caused by harmful factors like hepatotoxin in NAFLD patients, appropriate intervention should be applied. We studied the effect of JZG, which has been confirmed to improve liver function and to decrease liver fat accumulation and inflammation in our previous studies [19, 20]. In this study, JZG could decrease the inflammation and necrosis in liver tissues, and decrease liver transaminases, which had been increased in the group of HFD + CCl_4_, showing the effect of JZG on decreasing the sensitivity to hepatotoxin induced liver injury in NAFLD rats. In addition, the expression and activation of ERS related signaling molecues GRP78/PERK/EIF2α/NFκB, which had been obviously increased in HFD + CCl_4_ group, was down-regulated by JZG, suggesting that regulating ERS is part of the effective mechanism to improve the sensitivity of liver injury in NAFLD (Fig. 8). JZG is a herbal formula including a great deal of compounds. Our previous study has shown that the compounds derived from JZG such as protopanaxadiol, tanshinone IIA, and emodin have the effect of down-regulating lipid accumulation and oxidative stress in FFA indcuced hepatocytes. Wether these compounds or other bioactive components in JZG could contribute to the role of JZG in improving the liver injury of NAFLD through regulationg ERS requires further research [32].

## Conclusion

In summary, the present study demonstrated that HFD induced NAFLD rat was sensitized to hepatotoxic injury, which could be improved by JZG significantly. Regulation of ERS signaling pathway GRP78/PERK/EIF2α/NF-κB may represent part of the underlying pharmacological mechanism of JZG.

## Data Availability

All data generated or analyzed during this study are included in this article.

## Abbreviations

ALB: Albumin
ALT: Almandine Aminotransferase
AST: Aspartate Aminotransferase
EIF2α: Eukaryotic translation initiation factor 2α
ERS: Endoplasmic reticulum stress
GRP78: Glucose regulating protein
HDL-c: High density lipoprotein cholesterol
IRE1α: Inositol requiring enzyme 1α
JZG: Jiang-Zhi Granules
LDL-c: low density lipoprotein cholesterol
NAFLD: Non-alcoholic fatty liver
NASH: Non-alcoholic steatohepatitis
NFκB: Nuclear factor kappa B
PERK: Protein kinase RNA-like endoplasmic reticulum kinase
TC: Total cholesterol
TCM: Traditional Chinese medicine
TG: Triglyceride
TRAF2: Tumor necrosis factor receptor associated factor 2
γ-GT: γ-Glutamine transpetidase
